# Comparative transcriptome analysis of Parkinson’s disease and Hutchinson-Gilford progeria syndrome reveals shared susceptible cellular network processes

**DOI:** 10.1186/s12920-020-00761-6

**Published:** 2020-08-18

**Authors:** Diana M. Hendrickx, Enrico Glaab

**Affiliations:** Luxembourg Centre for Systems Biomedicine (LCSB), University of Luxembourg, 6, avenue du Swing, Belvaux, L- 4367 Luxembourg

**Keywords:** Parkinson’s disease, Aging, Hutchinson Gilford progeria syndrome, Network analysis, Meta-analysis

## Abstract

**Background:**

Parkinson’s Disease (PD) and Hutchinson-Gilford Progeria Syndrome (HGPS) are two heterogeneous disorders, which both display molecular and clinical alterations associated with the aging process. However, similarities and differences between molecular changes in these two disorders have not yet been investigated systematically at the level of individual biomolecules and shared molecular network alterations.

**Methods:**

Here, we perform a comparative meta-analysis and network analysis of human transcriptomics data from case-control studies for both diseases to investigate common susceptibility genes and sub-networks in PD and HGPS. Alzheimer’s disease (AD) and primary melanoma (PM) were included as controls to confirm that the identified overlapping susceptibility genes for PD and HGPS are non-generic.

**Results:**

We find statistically significant, overlapping genes and cellular processes with significant alterations in both diseases. Interestingly, the majority of these shared affected genes display changes with opposite directionality, indicating that shared susceptible cellular processes undergo different mechanistic changes in PD and HGPS. A complementary regulatory network analysis also reveals that the altered genes in PD and HGPS both contain targets controlled by the upstream regulator *CDC5L*.

**Conclusions:**

Overall, our analyses reveal a significant overlap of affected cellular processes and molecular sub-networks in PD and HGPS, including changes in aging-related processes that may reflect key susceptibility factors associated with age-related risk for PD.

## Background

Parkinson’s disease (PD) is one of the most common neurodegenerative disorders, with approximately 10 million affected persons worldwide [1]. Despite major advances in understanding PD genetics, no preventive or disease-modifying therapy is available [2]. Several studies have linked PD with aging-related cellular processes [3–5], showing that PD and aging share molecular hallmarks such as neuroinflammation [6], impaired DNA repair [7] and mitochondrial dysfunction [8]. Furthermore, PD has been hypothesized to represent an accelerated or premature form of aging, due to molecular changes that resemble aging-associated alterations but progress faster and/or occur earlier [3, 9, 10].

Among other aging-related disorders, Hutchinson–Gilford progeria syndrome (HGPS) at first sight does not resemble PD. As opposed to PD, HGPS mainly affects children and involves symptoms such as growth delay, short height, small face and hair loss [11], differing substantially from the typical motor- and non-motor symptoms observed in PD. However, previous studies have shown that many of the features associated with HGPS reflect a premature onset of pathologies commonly associated with adult aging and age-related neurodegenerative diseases [12, 13]. These observations suggest that a more systematic investigation of shared molecular alterations or shared susceptibility factors in PD and HGPS could provide new insights on a subset of generic, aging-associated pathological changes in PD that may already influence the early, pre-motor stages of the disease.

Most of the prior research on the molecular changes in PD or HGPS has focused on the analysis of transcriptomics data from a single study, e.g. PD brain microarray gene expression datasets from the *substantia nigra* midbrain region [14–20] and HGPS gene expression data from human fibroblasts [21–23]. However, to the best of our knowledge, an integrated meta-analysis and direct comparative investigation of molecular high-throughput data for PD and HGPS has not been conducted so far. Here, to address this gap we have applied independent meta-analyses for public PD and HGPS case-control transcriptomic datasets and then compared the aggregated statistics for the two diseases to identify significant shared variations at the level of single genes, pre-defined gene sets and local molecular subnetworks. For this purpose, we have interlinked differential expression meta-analyses with subsequent comparative pathway, network and co-expression analyses, assessing the significance of the overlap between PD and HGPS for each analysis type.Several methods for microarray meta-analysis have been developed [24–26], which can be divided into five categories. A first category covers methods that directly merge the raw data [27, 28]. A drawback of these methods is that systematic differences between studies often cannot be completely removed [25]. A second type of approaches combines effect sizes across studies. This approach may be suitable in particular when the effect size is the main statistic of interest. An example is the random effects model (REM) [29], implemented in the R Bioconductor package GeneMeta [30]. A third category combines ranks of differentially expressed genes. A representative approach is the Rank Product method, implemented in the RankProd Bioconductor package, which ranks the genes in each data set based on their fold change (FC) and combines the ranks by calculating their product [31]. A fourth type of methods involves the computation of latent variables, i.e. variables inferred using models from observed data. An example is the probability of expression (POE), implemented in the R Bioconductor metaArray package [32]. Finally, a further category of methods combines significance scores. These approaches may be preferred in particular when the *p*-value significance is the main statistic of interest. Examples are Fisher’s method [33] and Stouffer’s method [34] implemented in the metaDE R package, the combined *p*-value methods for paired and unpaired data in the metaMA R package [35], and the weighted meta-analysis method by Marot and Mayer [36] used in this study because of the sensitive combined *p*-value estimates it provides.

When performing a comparative analysis of two or more diseases, one has to take into account that differentially expressed genes (DEGs) potentially arising from alterations of generic processes can be detected in unrelated conditions [37]. Therefore, we included another aging-related disease (Alzheimer’s disease, AD) and another unrelated disease (primary melanoma, PM) as disorder controls to confirm that the observed overlapping affected genes and processes are non-generic.

Crow et al. [37] introduced the differential expression (DE) prior as a measure for a gene’s prior probability of being a DEG. The lower the DE prior, the higher the probability that a DEG is non-generic. By ranking a list of DEGs by their DE prior, candidate non-generic genes of interest for further investigation can be selected.

In summary, the comparative analysis of PD and HGPS data presented here extends beyond previous studies by: (1) providing a first systems-level statistical comparison of molecular changes in PD and HGPS derived from robust meta-analyses, and (2) revealing significant shared affected molecular factors in PD and HGPS at the level of individual genes, pre-defined gene sets and molecular subnetworks, which could pave the way towards the identification of new susceptibility genes and processes for early aging-associated pathological changes in PD.

## Methods

**Fig. 1:**
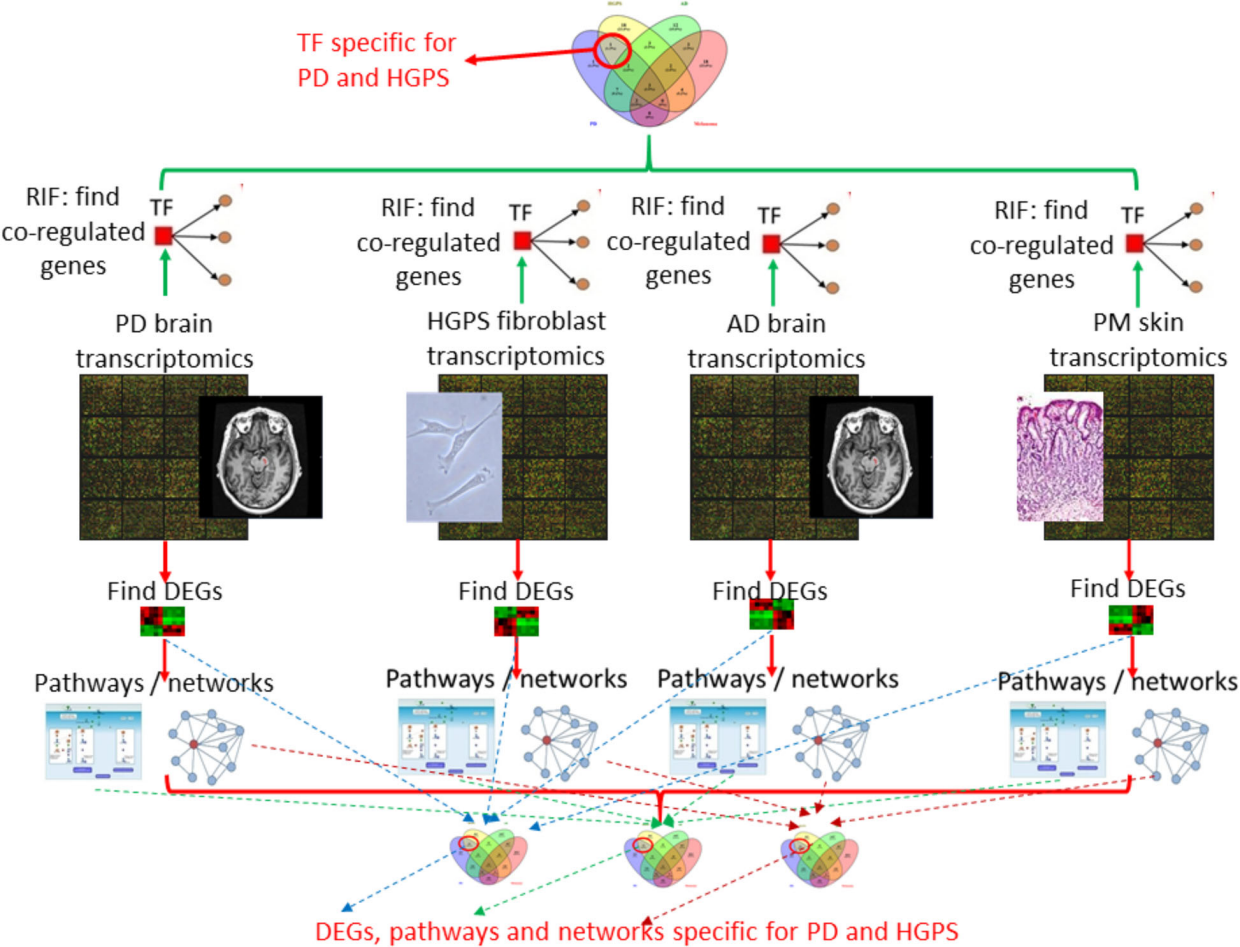
Overview of the workflow for the integrated meta-analysis of molecular high-throughput data for PD and HGPS, with AD and PM as control conditions. DEGs: differentially expressed genes; TF: transcription factors; RIF: regulatory impact factor analysis

The overal workflow of the statistical analysis procedures is depicted in Fig. 1. Since the available PD, HGPS, AD and PM data sets cover different disease conditions and are derived from different tissues, they were analyzed via separate meta-analyses. First, after pre-processing the transcriptomics data, differentially expressed genes (DEGs) between cases and controls were derived independently for each data set and, subsequently, a separate meta-analysis was conducted for each disease. Second, the lists of DEGs for each disease were further explored using cellular pathway and network analyses. Third, for each disease, key transcription factors (TFs) undergoing co-ordinated expression changes with their downstream target genes were determined by applying a co-expression analysis using the Regulatory Impact Factor (RIF) analysis approach and the TF-to-target pairs from UCSC (http://genome.ucsc.edu/). For each disease, the normalized expression data of the common genes across all datasets for the disease were combined, and the combined data set was used as input for the RIF analysis.

For all analysis types, the intersections among the results for the four diseases were determined. Only significant DEGs, pathways, networks and TFs only observed for PD and HGPS, but not significant for any of the other two diseases, were selected for further biological interpretation.

Although the main affected tissues differ between PD and HGPS, both disorders are characterized by a strong genetic component (HGPS is caused by the lamin A (*LMNA*) gene [38] and the total heritability of idiopathic PD has been estimated to be at least 0.27 [39]), suggesting that if their genetic susceptibility factors influence gene expression levels in overlapping pathways related to cellular aging, shared significant expression variation affecting these pathways can be identified across the expressed genes for different tissue types.

Given the strong genetic component in both diseases, we hypothesize that there are shared genetic susceptibility factors that result in a subset of transcript expression alterations in patients compared to controls which are independent of the age and the tissue context.

### Data sets for meta-analyses

Microarray gene expression data for PD, HGPS, AD and PM were collected from public case-control studies (see data source information in the section “Availability of data and materials”). For PD, the samples originate from *post mortem* biospecimens from the *substantia nigra* midbrain region. Samples for HGPS were derived from human cultured dermal fibroblasts. For AD, samples were taken from *post mortem* biospecimens from the hippocampus. PM case-control studies included skin samples from PM and normal skin. In order to pre-process all data using the same procedure, only Affymetrix microarray data sets for which the raw.CEL files were available were collected. In total, we extracted 11 data sets on PD, 3 on HGPS, 3 on AD and 2 on PM (see Table S1).

### Pre-processing and quality control

The Single-Channel Array Normalization (SCAN) pre-processing procedure [40], implemented in the SCAN.UPC package (version 2.24.1) from Bioconductor [41, 42], was applied on all microarray data sets for probe correction, normalization and removal of array-specific background noise. SCAN is a single-sample normalization method that adjusts for array type. Therefore, SCAN is particularly suited for integrative analyses of microarray data derived from different Affymetrix array platforms [40].

Quality control of all raw and pre-processed microarray data was conducted using the arrayQualityMetrics package (version 3.38.0) [43] from Bioconductor.

### Differential expression analyses

Before conducting differential expression analyses, the data were checked for covariates that could influence subsequent analyses. Significance of continuous and categorical covariates was determined using the t-test and Fisher’s exact test, respectively.

Differential gene expression analyses were applied at the probeset level to each dataset separately using the empirical Bayes moderated t-statistic [44] implemented in the Bioconductor limma package (version 3.38.3) [45], correcting for confounding covariates. Probes were mapped to genes using Bioconductor annotation packages (see Table S3 for an overview of annotation packages used in this study). Data for probes not corresponding to a gene were filtered out. In case multiple probes were assigned to the same gene, the probe with the highest absolute average expression level was chosen as the representative probe for that gene, since measurements from probes with lower average expression levels are less reliable. Nominal *p*-values of all PD (resp. HGPS, AD, PM) datasets were combined using the weighted meta-analysis method by Marot and Mayer [36]. This method uses weights for the number of samples in each data set to calculate a combined *p*-value. Next, the resulting combined *p*-values per gene were adjusted for multiple hypothesis testing using the Benjamini-Hochberg procedure [46], and a false discovery rate threshold of 0.05 was applied to select differentially expressed genes (DEGs). Because combination of *p*-values does not consider gene up/down regulation direction of each individual study, we applied an additional filtering step by selecting the genes that change consistently across all data sets, and only considered the selected genes for further analyses.

We then compared the obtained lists of DEGs via Venn diagrams using the web-application Venny [47]. To determine the significance of the overlap between two lists of DEGs, Fisher’s Exact test was applied.

### Gene set analysis

Alterations in the activity of pathways and biological processes were investigated using the software tool GeneGO MetaCore^TM^ (https://portal.genego.com/). Output tables from the differential expression analyses for PD, HGPS, AD and PM were used as input, including the adjusted *p*-value and median log fold change across all PD, HGPS, AD and PM data sets, respectively. To determine the top-ranked list of DEGs an adjusted *p*-value threshold of 0.05 was used. Apart from the *p*-value threshold, no further log fold change threshold was applied, in order to ensure that potentially relevant small-effect size changes in transcription factors with significant *p*-values are incorporated into the pathway analysis. Based on the gene table, GeneGO MetaCore^TM^ derives lists of significantly altered network objects, where genes are represented by the proteins they encode. For each of the four diseases, the list of DEGs was mapped onto GeneGO MetaCore^TM^’s canonical pathway maps and GO processes. To determine the enrichment of the top-ranked network objects in a particular canonical pathway map or GO process, GeneGO MetaCore^TM^ enrichment analysis applies the hypergeometric distribution test. In all analyses, *p*-values were corrected for multiple hypothesis testing using the false discovery rate approach by Benjamini and Hochberg [46].

The resulting lists of significantly altered canonical pathways and GO biological processes for the four diseases were compared via Venn diagrams. Pathways and GO processes significantly altered in PD and HGPS, but not in the other two diseases, were selected for further biological interpretation.

The list of significant processes only observed for PD and HGPS was further summarized and visualized using the web server REVIGO [48]. REVIGO forms clusters of highly similar GO terms for a user-provided similarity measure and a cut-off value for the similarity. In this study, the default settings using the simRel similarity measure of Schlicker et al. [49] and a similarity cut-off of 0.7 were used.

### Network analysis

In addition to the gene set analyses, GeneGO MetaCore^TM^ network analyses were applied to the lists of DEGs for the four diseases. In contrast to the gene set analysis, network analysis does not use pre-defined gene sets, but maps complete gene-level statistics to a genome scale protein-protein interaction network. This procedure identified multiple significantly altered molecular sub-networks for each of the diseases.

Here, we used the default “Analyze network“ algorithm in GeneGO MetaCore^TM^, with the maximum number of nodes in a sub-network limited to 50. This procedure determines the local altered molecular sub-networks surrounding the network objects from the input gene list as seed nodes using molecular interaction data and canonical pathway information from the GeneGO MetaCore^TM^ database. First, the lists of DEGs were mapped to their gene products (proteins, protein complexes). Then the gene products of the DEGs were connected with the proteins or protein complexes that have the highest connectivity with these gene products in the genome-scale protein-protein interaction network. This step is repeated iteratively until (maximum 30) sub-networks with a maximum of 50 nodes have been built (default). The sub-networks can have overlapping nodes, but no overlapping edges.

The lists of molecular sub-networks for the four diseases were compared using Venn diagrams, and only networks significantly altered in PD and HGPS, but not in the other two diseases, were selected for further biological interpretation.

### Regulatory impact factor analysis

In order to study potential shared upstream regulators for the four diseases, transcription factors (TFs) undergoing co-ordinated expression changes with the downstream target genes were determined from the collected microarray datasets using a Regulatory Impact Factor (RIF) analysis [50]. For each disease, the normalized expression data of the genes in all available data sets were combined into a single table and used as input for the RIF analysis. The RIF analysis was applied using the RIF implementation in the DCGL R-package (version 2.1.2) [51]. Prior to the computation of RIF scores, a gene filtration step was applied, filtering out genes with a Between-Experiment Mean Expression (BEMES) lower than the median of the BEMES for all genes and the genes that are not significantly more variable than the median gene, using a *p*-value threshold of 0.05. RIF scores were then determined on each of the four filtered lists using the current 199,950 TF-to-target interaction pairs from UCSC (http://genome.ucsc.edu). The resulting lists of TFs were compared via Venn diagrams, and the significance of the overlap between two lists of TFs was assessed using Fisher’s Exact test. Only TFs shared between PD and HGPS, but not significant for any of the other two control diseases were selected for further biological interpretation.

## Results

### Differential expression analyses

For each data set, Table S2 shows the clinical and demographic factors which were found significantly different between cases and controls based on a Fischer’s exact test (categorical variables) or a t-test (continuous variables). A correction for these confounders was applied in the differential expression analyses.

When conducting differential expression analysis on each data set separately, we noticed that 53% of the genes changed in the opposite direction in data set GSE54282 as compared to the majority of the other data sets (see Table S4). Data set GSE54282 was also the data set including the smallest number of samples (only 6 samples in total), see Table S1. Therefore it was excluded before applying the meta-analysis on PD.

**Fig. 2:**
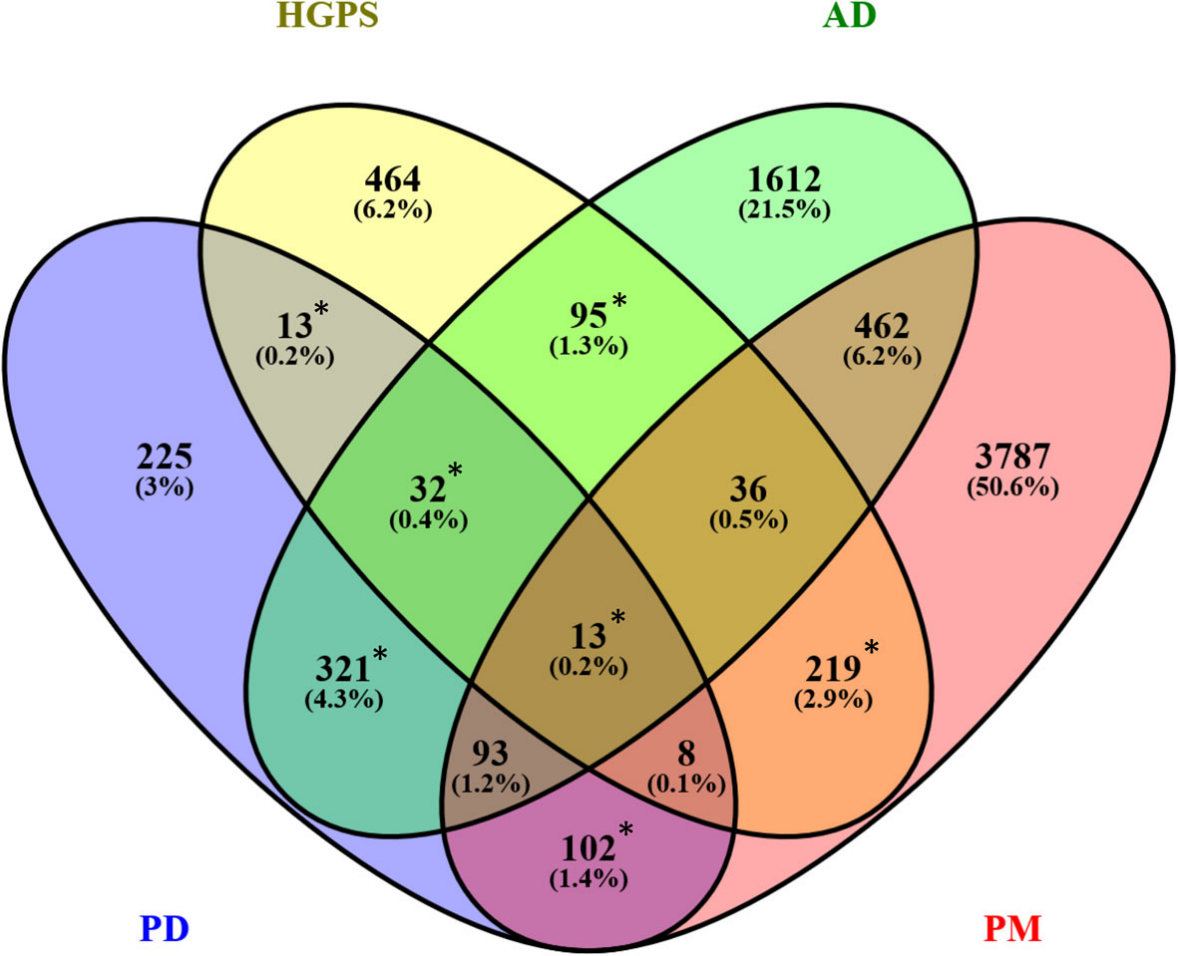
Shared significantly DEGs between PD, HGPS, AD and PM, determined using limma (adjusted *p*-value ≤0.05). *: significant overlap by Fisher’s exact test (*p*-value ≤0.05)

The differential expression analyses identified 807, 880, 2664 and 4720 DEGs for PD, HGPS, AD and PM respectively. When comparing disease-associated changes in PD and HGPS, 66 shared DEGs were identified (see Fig. 2), reflecting a significant overlap according to Fisher’s Exact test (*p*-value = 0.00026). From the 66 shared genes 13 were only observed for PD and HGPS, and not differentially expressed in any of the other two diseases. Table 1 shows the full name and the DE prior for these genes according to Crow et al. [37].

**Table 1:** DEGs found for PD and HGPS, but not for AD or PM

Direction in PD and HGPS	Gene symbol	Full name	DE prior
Opposite	CDH8	Cadherin 8	0.704543
	SRP19	Signal Recognition Particle 19	0.30116
	ARL3	ADP Ribosylation Factor Like GTPase 3	0.550125
	DNAJC12	DnaJ Heat Shock Protein Family (Hsp40) Member C12	0.969904
	RTL8C	Retrotransposon Gag Like 8C	0.567651
	NEDD8	NEDD8 Ubiquitin Like Modifier	n/a
	APOOL	Apolipoprotein O Like	0.665789
	CCR10	C-C Motif Chemokine Receptor 10	0.643647
	RABEPK	Rab9 Effector Protein With Kelch Motifs	0.136345
Same	KCNS3	Potassium Voltage-Gated Channel Modifier Subfamily S Member 3	0.782235
	CDH10	Cadherin 10	0.825788
	PTPRN	Protein Tyrosine Phosphatase Receptor Type N	0.683862
	DGKQ	Diacylglycerol Kinase Theta	0.361517

DE prior according to Crow et al. [37]. n/a: not reported

Of the 13 DEGs, 4 had the same fold change direction for PD and HGPS (*KCNS3*, *CDH10*, *PTPRN*, *DGKQ*)(Table 1). The other nine DEGs (*CDH8*, *SRP19*, *ARL3*, *DNAJC12*, *RTL8C*, *NEDD8*, *APOOL*, *CCR10*, *RABEPK*)(Table 1) changed in opposite directions in PD and HGPS, suggesting that different alterations may affect shared susceptibility genes in these disorders.

The 13 DEGs only found in PD and HGPS were compared with the 307 genes in the GenAge benchmark database of genes involved in aging (http://genomics.senescence.info/genes/index.html) [52], and none of the 13 genes was found in this database, suggesting that generic genes involved in aging were already removed by excluding genes involved in AD and PM.

### Gene set analysis

**Fig. 3:**
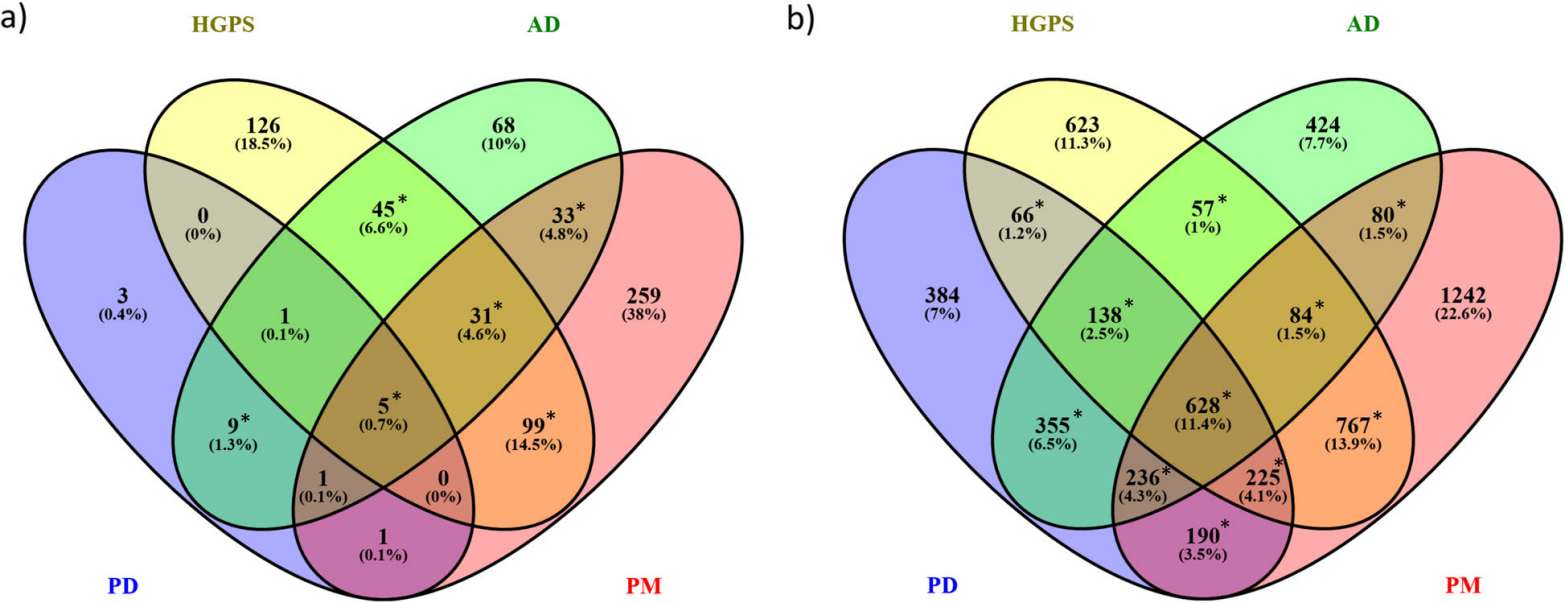
Shared significantly altered gene sets between PD, HGPS, AD and PM, determined using GeneGO MetaCore^TM^ enrichment analysis (adjusted *p*-value ≤0.05): **a**) shared canonical pathways; **b**) shared GO biological processes. *: significant overlap by Fisher’s exact test (*p*-value ≤0.05)

When applying GeneGO MetaCore^TM^ enrichment analysis on the list of DEGs for each disease, we identified 20, 307, 193 and 429 significantly altered pathways for PD, HGPS, AD and PM respectively. After determining the overlap of the results, we observed that 6 canonical pathways were shared between PD and HGPS (see Fig. 3a). However, all of these pathways were also significant for AD and five of them were for PM.

The GO analysis identified 2222, 2588, 2002 and 3452 significantly altered GO processes for PD, HGPS, AD and PM respectively. Furthermore, 1057 significantly altered GO biological processes were shared between PD and HGPS (see Fig. 3b). 66 of these GO processes were only observed for PD and HGPS, and were not significantly altered for any of the other two diseases. After summarizing the list of GO terms with REVIGO [48], the reduced list contained 48 GO biological processes. GO IDs, total size, directionality in PD and HGPS, and FDR for these GO terms are presented in Table S6.

### Network analysis

**Fig. 4:**
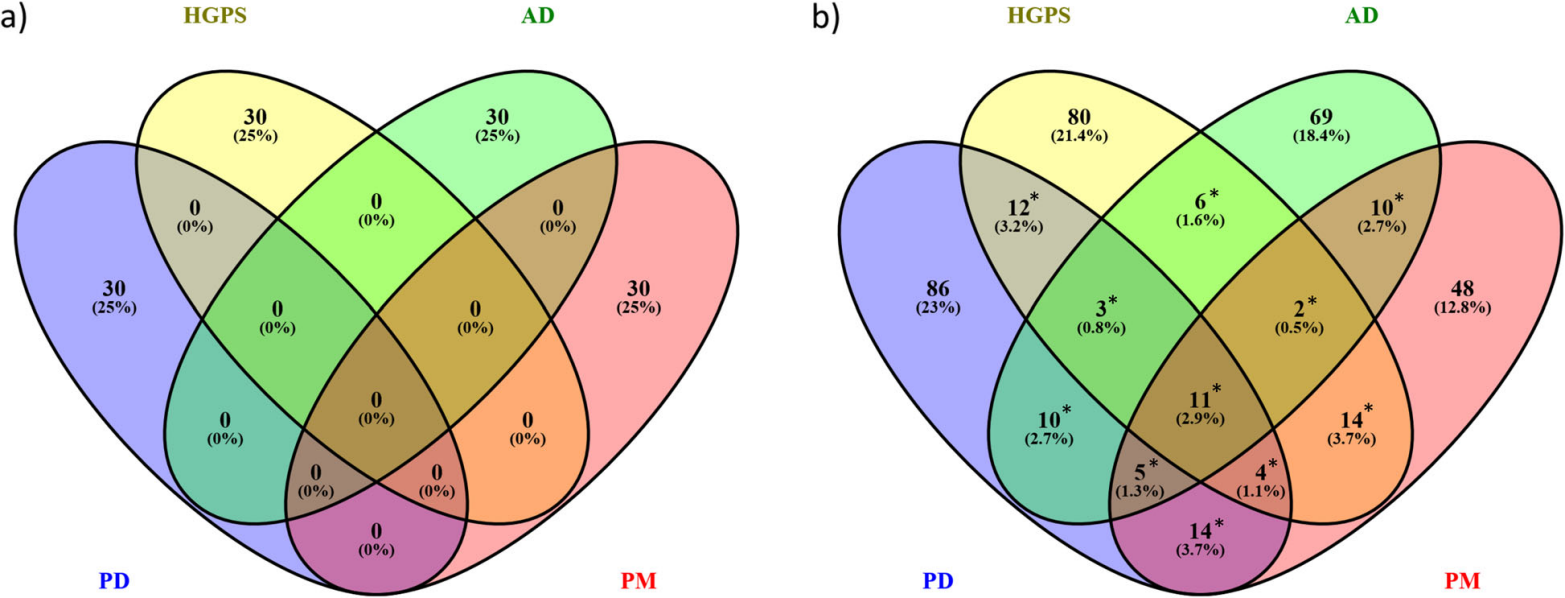
**a** Overlap of significantly altered subnetworks between PD, HGPS, AD and PM, determined using GeneGO MetaCore^TM^ network analysis. **b** Shared GO biological processes among the subnetworks for PD, HGPS, AD and PM. *: significant overlap by Fisher’s exact test (*p*-value ≤0.05)

When mapping the gene lists to a genome scale protein-protein interaction network using GeneGO MetaCore^TM^ network analysis, a maximum number of 30 sub-networks for each disease was identified, but the identified sub-networks show no overlap between any of the diseases (see Fig. 4a). The network analysis identified 145, 132, 116 and 108 GO-terms related to the sub-networks for PD, HGPS, AD and PM respectively, which partially overlap (see Fig. 4b). Twelve GO biological processes were associated with the sub-networks for PD and HGPS, but not with any of the other two diseases. For these 12 GO terms, Table 3 presents the key network objects of the sub-networks for PD and HGPS and the overlap with the seed nodes (gene products from the DEG lists) in these sub-networks. Moreover, the direction (up/down) of the alterations of these seed nodes is indicated.

### Regulatory impact factor analysis

**Fig. 5:**
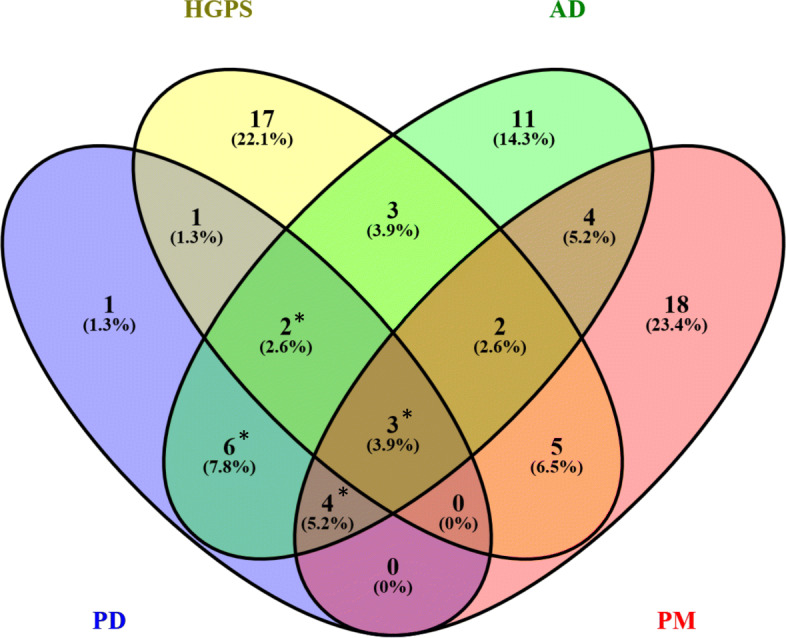
Overlap of key transcription factor alterations for PD, HGPS, AD and PM, determined using RIF analysis (*p*-value ≤0.05). *: significant overlap by Fisher’s exact test (*p*-value ≤0.05)

Apart from altered biological processes and subnetworks in PD and HGPS, we also identified changes in key regulatory genes, which can explain shared downstream variations. In particular, in order to find shared variations in key transcription factors (TFs), a Regulatory Impact Factor (RIF) analysis was conducted (see Methods). We identified 17, 33, 35 and 36 TFs for PD, HGPS, AD and PM respectively. In total, 6 shared TFs were found between PD and HGPS (see Fig. 5) and the overlap between the TFs for both diseases was statistically significant (*p*-value = 0.04, Fisher’s exact test). From the 6 shared TFs one (*CDC5L*) was only observed for PD and HGPS, and not identified in any of the two other diseases.

## Discussion

In this study we have presented the first transcriptome-wide comparison of expression changes in Parkinson’s disease (PD) and Hutchinson-Gilford Progeria Syndrome (HGPS) at the level of individual genes, cellular processes and molecular subnetworks. We included Alzheimer’s disease (AD) and primary melanoma (PM) as disorder controls to filter the results for overlapping, non-generic variations only observed for PD and HGPS, and performed robust case/control meta-analyses for each of the four diseases.

We identified 13 DEGs, 66 GO biological processes, 12 GO terms associated with molecular subnetworks and one TF with shared significance in PD and HGPS, and no significant alteration for the two control diseases.

### Shared DEGs only observed for PD and HGPS

We distinguish between two types of shared DEGs:

- *DEGs changing in the same direction in PD and HGPS*: these genes may serve for further investigation as candidate surrogate biomarkers for PD risk stratification and/or early diagnosis of PD;
- *DEGs changing in opposite direction*: these genes may represent shared susceptibility genes between the two diseases, which are altered by different disease-specific mechanisms.

To determine which of these genes are most likely non-generic DEGs, and therefore of particular interest for further study as shared susceptibility genes for PD and HGPS, we retrieved their DE prior from Crow et al. [37] (Table 1).

Among the 4 genes (*DGKQ*, *KCNS3*, *CDH10*, *PTPRN*) changing in the same direction, *DGKQ* has the lowest DE prior (0.36) and its deregulation is more likely to be non-generic and only occurring in PD and HGPS than the other 3 genes. *DGKQ* is one of the genes in the in the 4p16.3 region, which has been reported as one of the strongest PD risk loci by GWAS [53, 54], and has been associated with increased expression of *α*-synuclein [53]. Similarly, the second gene *KCNS3* was identified within a PD risk locus in a meta-analysis of Genome Wide Association Studies (GWAS) [55]. For the other two genes (*CDH10*, *PTPRN*) no PD- or HGPS-relevant information has been reported in previous studies.

For *NEDD8*, one of the genes changing in opposite direction for PD and HGPS, no DE prior is reported, which indicates that this gene was not differentially expressed in any of the 635 data sets analyzed by Crow et al. *NEDD8*, a gene associated with protein misfolding and aggregation, showed over-expression in progerin-induced aging in human induced pluripotent stem cells (iPSCs) [13]. Progerin is a truncated form of *LMNA*, the gene harboring mutations causing HGPS. Furthermore, a study in Drosophila suggests that impaired *NEDD8*-based modification of the PD-related proteins parkin and *PINK1* may contribute to PD pathogenesis [56]. Associations with PD are also corroborated by the observed accumulation of *NEDD8* in Lewy bodies in brain sections of PD patients [57].

Of the remaining 8 genes changing in opposite direction, *RABEPK*, a Rab9 effector protein has the lowest DE prior (0.14), and may therefore be of interest for further investigation as a candidate non-generic shared susceptibility gene only observed for PD and HGPS. Rab signaling has been implicated in PD due to the role of Rab proteins in intracellular vescicle trafficking [58].

Next, *CDH8* has been suggested to regulate dendritic spine morphogenesis based on rat experiments in the hippocampus [59]. Furthermore, experiments in human embryos have suggested that *CDH8* has a role in early cortical development [60]. *DNAJC12* plays an important role in biosynthesis and transport of dopamine, vesicle regeneration and protein folding [61]. In studies of unrelated families, mutations of *DNAJC12* have been associated with early-onset parkinsonism [62], dystonia and intellectual disability [63, 64].

A complete overview of references to further reported PD / HGPS associations for the identified 13 shared DEGs is provided in Supplementary Table S5.

### Potential mechanistic link between lamin a and neurodegeneration

Interestingly, *PPME1*, a gene previously linked to the HGPS-mutated gene lamin A (*LMNA*) [65], was significantly altered in both HGPS and the neurodegenerative disorders PD and AD, but not in the cancer disease PM. Dysregulation of *PPME1* has also been reported for the Parkinsonian age-related disorder Progressive Supranuclear Palsy by Park et al. [66]. *LMNA* is essential for *PP2A*-mediated dephosphorylations, which may be mediated by *PPME1* [65], which has been shown to limit the activity of *PP2A* by demethylating its catalytic subunit [67].

### Shared cellular process alterations only observed for PD and HGPS

The 66 shared GO biological processes only observed for PD and HGPS, identified by GeneGO MetaCore^TM^ enrichment analysis, tend to undergo alterations with different directionality (see Table S6). This suggests that the two diseases share multiple susceptibility-related processes, but these processes are perturbed through different mechanisms.

One of the identified clusters of robust shared significant GO terms (Table 2, cluster 1 Table S6) mainly contains processes related to movement of adaptive immune cells (helper T cells, CD8 cells). Interestingly, while adaptive immunity has been reported to be reduced during aging [68], these processes change in opposite direction in PD and HGPS (down in PD, up in HGPS).

**Table 2:** Clusters of shared significantly altered GO biological processes, determined by REVIGO (see Table S6)

Cluster representative	Direction of regulation	Keywords	Prior literature findings
regulation of intracellular calcium activated chloride channel activity	down in PD, up in HGPS	movement of adaptive immune cells	adaptive immunity reduced during aging [68]
regulation of peptidyl-threonine phosphorylation	down in PD, majority of genes down in HGPS (a few up)	DNA replication, telomere capping	genomic instability is a hallmark of aging [68] involved in premature aging and neurodegenerative diseases [12]
interleukin-8-mediated signaling pathway	cytokine pathways: down in PD, up in HGPS; adiponectin pathway: down in both PD and HGPS	cytokine signaling, adiponectin pathway	cytokine secretion in senescent cells during aging [68]
negative regulation of eosinophil activation	down in PD, up in HGPS	GPCR signaling, neuropeptide signaling	GPCRs in aging and PD [69]; GPCRs in (premature) cellular senescence [70]; neuropeptide in brain - neuromodulators [71]; neuropeptides in skin - wound healing [72]
response to axon injury	down in PD, majority of genes down in HGPS (a few up)	response to ROS	Increased levels of ROS related to aging [13, 68]
angiotensin-mediated vasodilation involved in regulation of systemic arterial blood pressure	down in PD, up in HGPS	blood pressure, metanephros (kidney)	None. GO terms only show up because of overlap with brain and skin processes.
regulation of cell projection assembly	down in PD, majority of genes down in HGPS (a few up)	cell projection organization	genes from cell projection GO terms as modifiers of neurodegeneration in PD [73]; cell projection in GO analysis of progerin-associated proteins [74]
viral genome replication	down in PD, majority of genes down in HGPS (a few up)	viral processes	Viral immunity decreases during aging [75]
myoblast development	down in PD, majority of genes down in HGPS (a few up)	nervous system development	PD might be associated with neurodevelopment [76]
morphogenesis of an endothelium	down in PD, majority of genes down in HGPS (a few up)	endothelial morphogenesis	application in neural stem cell-based therapy for PD [77]
circadian rhythm	down in PD, majority of genes down in HGPS (a few up)	circadian rhythm	Decrease of circadian rhythm associated with neurodegenerative disorders [78] and premature aging [79]
dopamine biosynthetic process	down in PD, up in HGPS	dopamine	dopamine in diagnosis and treatment of PD [80]; dopamine-specific processes in progerin-induced aging [13]
regulation of cytokinesis	down in PD, majority of genes up in HGPS (a few down)	cytokinesis	decrease of the number of cell divisions related to aging [81]
response to electrical stimulus	down in PD, majority of genes up in HGPS (a few down)	electrical stimulus	effect of electrical stimulation on gait in PD patients [82]
calcium-mediated signaling using intracellular calcium source	majority of genes down in PD and HGPS	calcium signaling	role in cellular senescence [83]; candidate hallmark of aging [68]; calcium signaling in premature aging [12]

**Table 3:** Shared GO processes between the subnetworks for PD and HGPS identified by the network analysis, but not related to subnetworks for AD or PM

GO term(s)	Network PD - key network objects	Seed nodes PD	Directionality PD	Network HGPS - key network objects	Seed nodes HGPS	Directionality HGPS
GO:0042320: regulation of circadian sleep/wake cycle, REM sleep	SLC18A1, NDUFB3, MPPED2, Matrilin 3, CELSR1	Galpha(q)-specific peptide GPCRs	down	GPR39, LCMT1, GLT25D2, BBS7, FAM126B	Galpha(q)-specific peptide GPCRs	both
GO:0022410: circadian sleep/wake cycle process	AKAP12, Neurocan, Alpha-internexin, CBARA1, PR61beta	Galpha(q)-specific peptide GPCRs, Galpha(i)-specific peptide GPCRs	down	RY1, p42 KKIALRE, ITM2A, Tho2, C11orf1	Galpha(q)-specific peptide GPCRs, Galpha(i)-specific peptide GPCRs	both
GO:0007076: mitotic chromosome condensation	CDK5, Doublecortin, SNAP19, ELOVL4, LDHD	none	n/a	GPR39, LCMT1, GLT25D2, BBS7, FAM126B	CAP-G, CAPG/G2	down
GO:0060024: rhythmic synaptic transmission	SDHC, M9, NDUFB6, AGAL, CACNA2D2	CACNA2D2, CACNA2D, Ca(II)channel R-type	down	VPS54, Rhophilin 2, RNF144, NDUFB6, FAM76B	none	n/a
GO:0006370: 7-methylguanosine mRNA capping, GO:0009452: 7-methylguanosine RNA capping	MLF1, CCK8, PGMU, OATP-E, FLJ22028	TBF5, TFIIH subunit	down, up	GSTM3, PDK (PDPK1), POLR2B, ATP6V0E2, C9orf16	POLR2B	down
GO:0014054: positive regulation of gamma-aminobutyric acid secretion	SNX4, LOH11CR2A, VPS41, MRPL46, ENDOGL1	none	n/a	MASP1, FUT8, Cubilin, SNX25, ARL13B	none	n/a
GO:0022900: electron transport chain	POLR3C, ATP1A4, nAChR beta-3, TMS-1, GATA-1	NDUFA2, NDUFA3	down	PPP1R3D, FOLR3, ERMAP, DNA polymerase sigma, WHDC1	none	n/a
GO:0070458: cellular detoxification of nitrogen compound	MANA, Rab-6B, LDB2, Exostosin-2, Rab-6	GSTM1, GSTs	down	TUB, PLEKHM2, ZNF507, SH3PXD2B, GATA-1	GSTs	up
GO:0007166: cell surface receptor signaling pathway	Tcf(Lef), PLC-beta, G-protein beta/gamma, MEK4 (MAP2K4), PKR	MAP3K1, p38alpha, GSK3-beta, TCF7L2(TCF4), Tcf(Lef)	down(3), up(2)	JAK1, Axin, Frizzled, Jagged1, LRP5	Dsh, SMAD3, TGF-beta 2, NOTCH1 receptor, FZD7, LRP6, NCOA3(pCIP/SRC3), RBP-J kappa(CBF1)	down(2), up(6)
GO:0032956: regulation of actin cytoskeleton organization	DCTN6, PGRMC1, SEZ6L2, SCN9A, Cdc42 subfamily	RHO6, Cdc42 subfamily, Rho GTPase	down	PQLC1, TSHZ1, ZNF317, Cdc42 subfamily, FAN	SPTA1, Rho GTPase, Rac1, Rho3	down(1), up(1), both(2)
GO:0007167: enzyme-linked receptor protein signaling pathway	Ephrin-A, H-Ras, P13K reg class IA, Ephrin-A5, RHO6	Ephrin-A receptor 8, H-Ras, Ephrin-A receptor 2	down	ERK1/2, c-Src, VEGFR-2, JAK1, Ephrin-A	Ephrin-B, Ephrin-A receptor 2, Ephrin-A2, Ephrin-A	down(3), up(1)

Each GO term is presented together with the subnetworks for PD and HGPS, shared seed nodes between the subnetwork and the GO processes, and regulation directionality of the seed nodes. n/a: not applicable

A second cluster of GO terms (Table 2, cluster 2 Table S6) includes the related terms “GO:0045740: positive regulation of DNA replication“ and “GO:1904353: regulation of telomere capping“. In HGPS, the majority of the genes within these processes show lower expression, while in PD all genes show lower expression. Genomic instability, the accumulation of DNA damage, is known as one of the hallmarks of aging [68], and thought to be involved in both premature aging and age-related neurodegenerative diseases [12].

Alterations are also observed in the regulation of cytokine signaling, including the chemokines interleukin-8 (IL-8 or CXCL8) and CXCR4, and the inflammatory cytokine macrophage migration inhibitory factor (MIF). The corresponding cluster (Table 2, cluster 3 Table S6) also covers the adiponectin-activated signaling pathway, which has been reported to modify cytokine expression in endothelial cells according to experiments in mouse brains [84]. While the majority of the genes in the adiponectin pathway show lower expression in both PD and HGPS, the cytokine pathways change in opposite directions (down in PD, up in HGPS). Secretion of pro-inflammatory cytokines has been observed in senescent cells, which are known to accumulate during aging [68].

A complete list of the clusters of shared significant GO term alterations is shown in Table 2.

### Shared cellular processes related to deregulated subnetworks only observed for PD and HGPS

The GeneGO MetaCore^TM^ network analysis identified 12 shared GO biological processes reflecting altered subnetworks for both PD and HGPS. Seed node DEGs associated with the same GO processes for PD and HGPS differ both in composition and, for the overlapping nodes, in the direction of the alteration, pointing to diverse mechanisms operating on functionally related sets of genes. Specifically, four processes showed a similar overlap of seed nodes, but are regulated in different expression directions for PD and HGPS (Table 3): “GO:0042320: regulation of circadian sleep/wake cycle, REM sleep“, “GO:0022410: circadian sleep/wake cycle process“, “GO:0070458: cellular detoxification of nitrogen compound“, “GO:0032956: regulation of actin cytoskeleton organization“ and “GO:0007167: enzyme-linked receptor protein signaling pathway“. Two of them are related to circadian rhythm, corresponding to the results of the gene set analysis. For PD, the seed nodes are regulated by genes which show lower expression, while for HGPS they are regulated by a combination of genes regulated in different directions (Table 3). A similar relationship also applies to the GO terms “GO:0032956: regulation of actin cytoskeleton organization“ and “GO:0007167: enzyme-linked receptor protein signaling pathway“. For the shared stress response “GO:0070458: cellular detoxification of nitrogen compound“, the seed nodes change in opposite directions in PD and HGPS (down in PD, up in HGPS, see Table 3).

Three processes show similar overlap of GO terms and subnetworks for PD and HGPS, but direct regulation through seed node genes is only observed for one of the diseases (either PD or HGPS) (Tables 3 and S7): “GO:0007076: mitotic chromosome condensation“, “GO:0060024: rhythmic synaptic transmission“ and “GO:0022900: electron transport chain“. These observations point to processes that are directly regulated by DEGs in one disease, but indirectly regulated in the other. Specifically, for the cell cycle process “GO:0007076: mitotic chromosome condensation“, an overlap is only observed between this GO term and the network neighborhood surrounding of the seed nodes for PD, whereas for HGPS the overlap contains seed node genes with decreased expression. Indeed, lower expression of cell cycle activity has been observed in stem cells of aging mice [81]. For PD, an overlap is observed between “rhythmic synaptic transmission (GO:0060024)“ and the seed nodes, whereas for HGPS there is only an overlap with the seed node neighborhood.

Similarly, the observed overlap between the subnetworks and the process “GO:0022900: electron transport chain“ includes seed nodes which are lower expressed in PD, whereas for HGPS, only nodes in the seed node neighborhood were present. Destabilization of the electron transport chain leads to mitochondrial dysfunction and the generation of reactive oxygen species (ROS), which has been associated with cellular aging [12, 68]. The response to ROS also occured among the significant processes in the gene set analysis.

All other shared processes (“GO:0006370: 7-methylguanosine mRNA capping“, “GO:0009452: 7-methylguanosine RNA capping“, “GO:0014054: positive regulation of gamma-aminobutyric acid secretion“ and “GO:0007166: cell surface receptor signaling pathway“) show a different overlap with the subnetworks for PD and HGPS (see Tables 3 and S8). In summary, our network analyses reveal significant shared biological processes between PD and HGPS that differ in regulation directionality, direct or indirect regulation by the DEGs or through the mechanisms by which they are regulated. These observations indicate that shared susceptible molecular subnetworks between PD and HGPS are modulated in a disease-specific manner.

### Shared key transcription factor (TF) alterations only observed for PD and HGPS

A shared altered TF only observed for PD and HGPS was identified in the RIF analysis: the spliceosome component *CDC5L*. Interestingly, this gene has previously been reported to contribute to increased chomosomal changes (aneuploidy) associated with the aging process [85].

### Shared susceptibility factors independent of age and tissue

The statistically significant overlaps between transcriptome alterations in PD and HGPS we observed lend further support to our hypothesis that there are shared genetic susceptibility factors which are independent of age and tissue. We acknowledge that further study will be needed to delineate the underlying genetic factors and corroborate the associated gene, pathway and network alterations that may be involved in conferring shared susceptibility.

### Comparison with other meta-analyses on PD and AD

Several other research groups have conducted meta-analyses on PD and/or AD. Kelly et al. performed a meta-analysis on public data sets for PD from the *substantia nigra*, using an approach that combines effect sizes [86]. They identified 1046 DEGs, of which 632 were measured in all PD data sets in our study. The 632 DEGs found by Kelly et al. have a significant overlap of 303 genes with the DEGs for PD found in our study (Fisher’s exact test *p*-value = 1.84e-151). Furthermore, they found an overlap of 436 DEGs with a previous meta-analysis on AD by Li et al. [87], of with 271 genes were measured in all PD and AD data sets in our study. There was a significant overlap of 108 genes of these 271 genes with the intersection of DEGs for PD and AD in our study (Fisher’s exact test *p*-value = 7.55e-65).

Li et al. [87] conducted a meta-analysis on AD using the same combined *p*-value approach as in our study, but collected public data sets originating from the frontal cortex instead of the hippocampus. They found 3124 DEGs, of which 2586 were measured in all AD data sets in our study. These 2586 DEGs show a significant overlap of 728 genes with the DEGs from AD in our study (Fisher’s exact test *p*-value = 6.14e-70).

Su et al. performed a meta-analysis on five public PD data sets from the *substantia nigra* by determining the intersection of the DEGs from the five individual data sets, and identified 17 common DEGs [88]. Three of these genes were also DEGs for PD in our study, 2 of them were not measured in all PD data sets in our study, and the remaining 12 were not differentially expressed in our study, which was based on twice as many data sets as the study of Su et al.

Zheng et al. applied the combined *p*-value method from the R package metaMA to conduct a meta-analysis on three public data sets on AD from the hippocampus and compared their results with those of a data set on normal aging [89]. They found 6205 DEGs for the AD meta-analysis, of which 1291 were also found for normal aging. They did not report the full list of 1291 genes, but only the top 50. Of these top 50 genes, 47 were measured for all AD data sets in our study. Of these 47 genes, 31 were also DEGs for AD in our study, of which 12 occurred in AD only, 13 in AD and PD but not in HGPS, 3 in AD and HGPS but not in PD, and 3 in all of the aging-related diseases.

Moradifard et al. conducted a meta-analysis on 6 datasets for AD from various brain tissues using the ranking-based approach from the R package RobustRankAggreg [90]. They identified 1404 DEGs, of which 1218 were measured in all AD data sets in our study. These 1218 DEGs displayed a significant overlap of 413 DEGs with those found in our study (Fisher’s exact test *p*-value = 2.37e-60).

### Limitations of this study

In order to enable pre-processing of all data sets using the same procedure, only Affymetrix microarray data sets for which the raw.CEL files were publicly available were collected.

Another shortcoming related to data availability concerns the meta-data that is shared together with the microarray data sets, which differs between studies. Availability of sufficient meta-data is important to check for an influence of potential confounding factors in the clinical and demographic data.

The study focused on a single key affected tissue per disease, hence, the outcome would differ if data from another affected tissue had been chosen. However, the comparison with other meta-analyses above shows that results from meta-analyses in different tissues display a significant overlap.

Finally, as the meta-analysis approach used in this study is based on combining *p*-values, the results are limited to genes that were measured in all data sets for the studied disease. However, an advantage of the weighted *p*-value approach is that, in contrast to the majority of other meta-analysis methods, this method can take into account the size of the different data sets, and in this way assigns more weight to data sets with larger sample sizes.

## Conclusions

Parkinson’s Disease (PD) and Hutchinson-Gilford Progeria Syndrome (HGPS) are both disorders associated with the aging process, which had not yet been compared at a molecular level. Although different tissues are affected in these diseases, a molecular-level comparison is justified by the fact that genetic alterations, with potential shared aging-associated susceptibility factors, play an important role in both disorders. Here, we have conducted a transcriptome-wide comparison, including Alzheimer’s disease (AD) and primary melanoma (PM) as control diseases. Overall, the integrative analysis revealed significant shared alterations at all the investigated scales (single gene, gene set and network level) and identified a shared non-generic change in a key transcription factor (*CDC5L*), correlating with downstream expression changes for both PD and HGPS.

When studying the non-generic shared significant genes at the level of gene set and network alterations, the results indicate that the two diseases undergo different mechanistic alterations, but that these alterations often operate on the same susceptible cellular processes. In line with previously known associations of the two disorders with aging, several of the molecular changes affect age-related cellular processes, e.g. DNA damage response, ROS signaling, cell cycle activity and mitochondrial dysfunction. In particular, shared processes previously implicated in premature aging (decreased circadian rhythm, calcium signaling) were identified. Interestingly, expression alterations linked with developmental and morphogenic processes were also observed.

Since HGPS is characterized by a premature onset of cellular pathologies resembling those in age-related neurodegenerative diseases, such as PD, the significant shared transcriptomic changes in PD and HGPS identified here may coincide with a subset of susceptibility-associated genes and processes which may be involved in mediating the effects of cellular aging on PD. Follow-up studies will need to extend these analyses to longitudinal expression profiling experiments and measurements in atypical forms of Parkinsonism and other neurodegenerative disorders in order to better understand the time-dependence and specificity of deregulations in these aging- and PD-associated processes.

## Supplementary information

**Additional file 1** Supplementary material. Supplementary tables.

## Data Availability

The datasets analysed during the current study, and listed in Table S1, are available in the Gene Expression Omnibus (GEO)(https://www.ncbi.nlm.nih.gov/geo/) and ArrayExpress (https://www.ebi.ac.uk/arrayexpress/) repositories. The accession numbers and weblinks to these data sets are listed below. Links to data sets on Parkinson’s disease in the *Substantia Nigra*: • GSE49036: https://www.ncbi.nlm.nih.gov/geo/query/acc.cgi?acc=GSE49036 • GSE54282: https://www.ncbi.nlm.nih.gov/geo/query/acc.cgi?acc=GSE54282 • GSE20163: https://www.ncbi.nlm.nih.gov/geo/query/acc.cgi?acc=GSE20163 • GSE20164: https://www.ncbi.nlm.nih.gov/geo/query/acc.cgi?acc=GSE20164 • GSE20292: https://www.ncbi.nlm.nih.gov/geo/query/acc.cgi?acc=GSE20292 • GSE20333: https://www.ncbi.nlm.nih.gov/geo/query/acc.cgi?acc=GSE20333 • GSE8397: https://www.ncbi.nlm.nih.gov/geo/query/acc.cgi?acc=GSE8397 • GSE7621: https://www.ncbi.nlm.nih.gov/geo/query/acc.cgi?acc=GSE7621 • GSE20141: https://www.ncbi.nlm.nih.gov/geo/query/acc.cgi?acc=GSE20141 • GSE7307: https://www.ncbi.nlm.nih.gov/geo/query/acc.cgi?acc=GSE7307 The data from Simunovic et al. [20] were obtained from the National Brain Databank at the Harvard Brain Tissue and Resource Center/McLean Hospital (https://hbtrc.mclean.harvard.edu/), and are available from the original authors on reasonable request. Links to data sets on Hutchinson-Gilford progeria syndrome in skin fibroblasts: • GSE3860: https://www.ncbi.nlm.nih.gov/geo/query/acc.cgi?acc=GSE3860 • E-MEXP-2597: https://www.ebi.ac.uk/arrayexpress/experiments/E-MEXP-2597/ • E-MEXP-3097: https://www.ebi.ac.uk/arrayexpress/experiments/E-MEXP-3097/ Links to data sets on Alzheimer’s disease in the hippocampus: • GSE48350: https://www.ncbi.nlm.nih.gov/geo/query/acc.cgi?acc=GSE48350 • GSE36980: https://www.ncbi.nlm.nih.gov/geo/query/acc.cgi?acc=GSE36980 • GSE5281: https://www.ncbi.nlm.nih.gov/geo/query/acc.cgi?acc=GSE5281 Links to data sets on primary melanoma: • GSE15605: https://www.ncbi.nlm.nih.gov/geo/query/acc.cgi?acc=GSE15605 • GSE7553: https://www.ncbi.nlm.nih.gov/geo/query/acc.cgi?acc=GSE7553 The reference data set of TF-to-target interaction pairs used for the Regulatory Impact Factor (RIF) analysis in this study was extracted from the DCGL R-package [51] (https://cran.r-project.org/web/packages/DCGL/index.html, tf2target data) and includes 199,950 TF-to-target interactions from the University of California Santa Cruz (UCSC) Genome Browser (http://genome.ucsc.edu/). The GenAge benchmark database of genes related to ageing can be accessed via http://genomics.senescence.info/genes/index.html.

## Abbreviations

AD: Alzheimer’s disease
BEMES: Between-experiment mean expression
DE: Differential expression
DEGs: Differentially expressed genes
FC: Fold change
FDR: False discovery rate
GEO: Gene expression omnibus
GO: Gene Ontology
GWAS: Genome wide association studies
iPSCs: Induced pluripotent stem cells
HGPS: Hutchinson-Gilford Progeria syndrome
limma: Linear models for microarray data
PD: Parkinson’s disease
PM: Primary melanoma
POE: Probability of expression
REM: Random effects model
RIF: Regulatory Impact Factor
ROS: Reactive oxygen species
SCAN: Single-channel array normalization
TF: Transcription factor
