# Supplementary material for: Comparative transcriptome analysis of Parkinson’s disease and Hutchinson-Gilford progeria syndrome reveals shared susceptible cellular network processes
Source: BMC Med Genomics. 2020 Aug 18;13:114. doi: 10.1186/s12920-020-00761-6 (PMC7437934; doi:10.1186/s12920-020-00761-6)
Supplement: Supplementary file 1 — Additional file 1 Supplementary material. Supplementary tables. [file 12920_2020_761_MOESM1_ESM.pdf]

Supplementary tables

June 2, 2020

Supplementary tables

Table S1: Overview of data sets included in this study.

Disease	Platform	Conditions/samples	Reference/Database
PD	HG-U133 Plus 2	PD (15), control (8)	Dijkstra et al.(2015) [1], GEO:GSE49036
PD	HuGene-1.0-st	PD (3), control (3)	Riley et al.(2014) [2], GEO:GSE54282
PD	HG-U133A	PD (8), control (9)	Zheng et al.(2010) [3], GEO:GSE20163
PD	HG-U133A	PD (6), control (5)	Zheng et al.(2010) [3], GEO:GSE20164
PD	HG-U133A	PD (11), control (18)	Zhang et al.(2005) [4], GEO:GSE20292
PD	HG-Focus	PD (6), control (6)	GEO:GSE20333
PD	HG-U133A, HG-U133B	PD (24), control (15)	Moran et al.(2006) [5], GEO:GSE8397
PD	HG-U133 Plus 2	PD (16), control (9)	Lesnick et al.(2007) [6], GEO:GSE7621
PD	HG-U133 Plus 2	PD (10), control (8)	Zheng et al.(2010) [3], GEO:GSE20141
PD	HG-U133A	PD (10), control (9)	Simunovic et al.(2008) [7]
PD	HG-U133 Plus 2	PD (9), control (17)	GEO:GSE7307
HGPS	HG-U133A, HG-U133B	HGPS (9), control (9)	Csoka et al.(2004) [8], GEO:GSE3860
HGPS	HG-U133 Plus 2	HGPS (5), control (5)	Marji et al.(2010) [9], ArrayExpress:E-MEXP-2597
HGPS	HG-U133 Plus 2	HGPS (3), control (6)	Plasilova et al.(2011) [10], ArrayExpress:E-MEXP-3097
AD	HG-U133 Plus 2	AD (18), control (23)	Berchtold et al.(2008) [11], GEO:GSE48350
AD	HuGene-1.0-st	AD (7), control (10)	Hokama et al.(2014) [12], GEO:GSE36980
AD	HG-U133 Plus 2	AD (10), control (13)	Liang et al.(2007) [13], GEO:GSE5281
PM	HG-U133 Plus 2	PM (46), control (16)	Raskin et al.(2013) [14], GEO:GSE15605
PM	HG-U133 Plus 2	PM (16), control (4)	Riker et al.(2008) [15], GEO:GSE7553

Table S2: Confounding factors for each data set (clinical and demographic factors which are significantly different between cases and controls).

Disease	Data set	Confounders
PD	GSE49036	none
PD	GSE54282	none
PD	GSE20163	none
PD	GSE20164	none
PD	GSE20292	age (p-value t-test = 0.03279)
PD	GSE20333	none
PD	GSE8397	none
PD	GSE7621	none
PD	GSE20141	none
PD	Simunovic et al.	none
PD	GSE7307	none
HGPS	GSE3860	none
HGPS	E-MEXP-2597	none
HGPS	E-MEXP-3097	none
AD	GSE48350	none
AD	GSE36980	age (p-value t-test = 0.00060)
AD	GSE5281	none
PM	GSE15605	none
PM	GSE7553	none

Table S3: Bioconductor annotation packages used in this study.

Platform	Annotation package
HG-U133 Plus 2	hgu133plus2.db
HuGene-1.0-st	hugene10sttranscriptioncluster.db
HG-U133A	hgu133a.db
HG-U133B	hgu133b.db
HG-Focus	hgfocus.db

Table S4: Percentage of genes in each data set that do not change their expression in disease versus control consistently compared to the majority of the data sets.

data set	% of genes
GSE7307	25%
GSE7621	25%
GSE8397	23%
GSE20141	32%
GSE20163	30%
GSE20164	27%
GSE20292	25%
GSE20333	32%
GSE49036	28%
GSE54282	53%
Simunovic et al.	35%

Table S5: DEGs found for PD and HGPS, but not for AD or PM. Log fold changes in PD and HGPS (red: up, blue: down). Implications in PD and HGPS in the literature.

gene symbol	median logFC PD	median logFC HGPS	implications in PD in the literature	implications in HGPS in the literature
CDH8	-0.37402	0.13807	no	no
SRP19	-0.17529	0.05867	no	no
ARL3	-0.16196	0.09760	no	no
DNAJC12	-0.29735	0.13393	yes [16, 17]	no
RTL8C	-0.24840	0.05801	no	no
NEDD8	-0.16029	0.06917	yes [18, 19]	no
APOOL	-0.16038	0.06821	no	no
CCR10	-0.04835	0.13802	no	no
RABEPK	-0.0814	0.15783	no	no
KCNS3	-0.27141	-0.39726	yes [20]	no
CDH10	-0.20774	-0.20152	no	no
PTPRN	-0.21739	-0.21608	no	no
DGKQ	-0.05870	-0.15804	yes [21, 22, 23]	no

Table S6: Significantly altered GO terms found for both PD and HGPS, but not for AD or PM, grouped in clusters by REVIGO [24]. The number of network objects from the DEGs is shown in brackets.

cluster	GO term	total size	direction PD	direction HGPS	FDR PD	FDR HGPS
1	GO:1902938: regulation of intracellular calcium activated chloride channel activity	5	down (4)	up (2)	0.00016	0.04813
	GO:2000473: positive regulation of hematopoietic stem cell migration	4	down (2)	up (2)	0.03893	0.03322
	GO:2000662: regulation of interleukin-5 secretion	12	down (3)	up (2)/down (1)	0.04450	0.03739
	GO:0035705: T-helper 17 cell chemotaxis	3	down (2)	up (2)	0.02338	0.01965
	GO:2000458: regulation of astrocyte chemotaxis	4	down (2)	up (2)	0.03893	0.03322
	GO:2000451: positive regulation of CD8-positive, alpha-beta T cell extravasation	4	down (2)	up (2)	0.03893	0.03322
	GO:0035704: helper T cell chemotaxis	3	down (2)	up (2)	0.02338	0.01965
2	GO:0010799: regulation of peptidyl-threonine phosphorylation	68	down (10)	up (3)/down (6)	0.00324	0.00754
	GO:0045740: positive regulation of DNA replication	66	down (11)	up (3)/down (7)	0.00066	0.00179
	GO:0035306: positive regulation of dephosphorylation	94	down (12)	up (6)/down (4)	0.00343	0.01804
	GO:0033138: positive regulation of peptidyl-serine phosphorylation	166	down (16)	up (14)/down (3)	0.00854	0.00220
	GO:0090218: positive regulation of lipid kinase activity	64	down (10)	up (5)/down (9)	0.00209	2.55E-06
	GO:1904353: regulation of telomere capping	33	down (5)	up (1)/down (5)	0.04198	0.00872
3	GO:0038112: interleukin-8-mediated signaling pathway	4	down (2)	up (2)	0.03893	0.03322
	GO:0038160: CXCL12-activated CXCR4 signaling pathway	3	down (2)	up (2)	0.02338	0.01965
	GO:2000448: positive regulation of macrophage migration inhibitory factor signaling pathway	4	down (2)	up (2)	0.03893	0.03322
	GO:0033211: adiponectin-activated signaling pathway	11	down (3)	up (1)/down (3)	0.03804	0.00398
	GO:0038159: C-X-C chemokine receptor CXCR4 signaling pathway	3	down (2)	up (2)	0.02338	0.01965
4	GO:1902567: negative regulation of eosinophil activation	4	down (2)	up (2)	0.03893	0.03322
	GO:0060160: negative regulation of dopamine receptor signaling pathway	5	down (3)	up (1)/down (1)	0.00451	0.04813
	GO:0038178: complement component C5a signaling pathway	4	down (2)	up (2)	0.03893	0.03322
	GO:0090663: galanin-activated signaling pathway	4	down (2)	up (2)	0.03893	0.03322
5	GO:0048678: response to axon injury	146	down (15)	up (6)/down (11)	0.00654	0.00058
	GO:0034614: cellular response to reactive oxygen species	254	down (26)	up (5)/down (22)	0.00266	0.00004
	GO:0071315: cellular response to morphine	17	down (4)	up (1)/down (3)	0.02192	0.01736
	GO:0038166: angiotensin-activated signaling pathway	19	down (4)	up (2)/down (3)	0.02998	0.00427
6	GO:0002033: angiotensin-mediated vasodilation involved in regulation of systemic arterial blood pressure	4	down (2)	up (3)	0.03893	0.00173
	GO:0035566: regulation of metanephros size	4	down (2)	up (2)	0.03893	0.03322
	GO:0035932: aldosterone secretion	3	down (2)	up (2)	0.02338	0.01965
	GO:0072216: positive regulation of metanephros development	21	down (4)	up (3)/down (1)	0.03907	0.03251
7	GO:0060491: regulation of cell projection assembly	299	down (28)	up (13)/down (17)	0.00046	0.00004
	GO:0031274: positive regulation of pseudopodium assembly	21	down (4)	up (2)/down (2)	0.03907	0.03251
	GO:1902743: regulation of lamellipodium organization	75	down (12)	up (2)/down (12)	0.00050	0.00002
	GO:0051491: positive regulation of filopodium assembly	54	down (10)	up (4)/down (7)	0.00057	0.00008
8	GO:0019079: viral genome replication	43	up (1)/down (5)	up (1)/down (5)	0.03461	0.02647
	GO:0019048: modulation by virus of host morphology or physiology	65	down (8)	up (5)/down (5)	0.02372	0.00162
	GO:0050792: regulation of viral process	330	up (2)/down (23)	up (10)/down (17)	0.01405	0.00215
9	GO:0048627: myoblast development	4	down (2)	up (1)/down (1)	0.03893	0.03322
	GO:0021953: central nervous system neuron differentiation	308	up (2)/down (23)	up (11)/down (15)	0.00647	0.00177
	GO:0022029: telencephalon cell migration	100	down (13)	up (4)/down (10)	0.00184	0.00036
	GO:0021885: forebrain cell migration	103	down (13)	up (4)/down (11)	0.00237	0.00014
10	GO:0003159: morphogenesis of an endothelium	19	down (5)	up (4)/down (2)	0.00551	0.00057
	GO:0060661: submandibular salivary gland formation	4	down (2)	down (2)	0.03893	0.03322
11	GO:0007623: circadian rhythm	281	down (22)	up (10)/down (15)	0.01625	0.00113
12	GO:0042416: dopamine biosynthetic process	18	down (7)	up (4)	0.00005	0.02034
13	GO:0032465: regulation of cytokinesis	121	down (13)	up (7)/down (5)	0.00879	0.01440
14	GO:0051602: response to electrical stimulus	117	up (1)/down (12)	up (9)/down (8)	0.00672	0.00004
15	GO:0035584: calcium-mediated signaling using intracellular calcium source	29	up (2)/down (7)	up (2)/down (3)	0.00002	0.02163

Table S7: Network analysis - GO processes with similar overlap with the subnetworks for PD and HGPS, but direct regulation through seed node genes only observed for one of the diseases.

Process	Overlap with PD and HGPS networks
GO:0007076: mitotic chromosome condensation	CAP-H/H2, hCAP-H2, Condensin, GATA-1
GO:0022900: electron transport chain	COX II, COX III, Cytochrome-c oxidase, COX VIa, COX VIII-3, COX VIII, COX VIa-2, GATA-1
GO:0060024: rhythmic synaptic transmission	Ca(II)channel R-type, CACNA2D, CACNA2D2, GATA-1

Table S8: Network analysis - shared GO processes with a different overlap with the subnetworks for PD and HGPS.

Process	Observations
GO:0006370: 7-methylguanosine mRNA capping	Only two gene products in common: POLR2B and GATA-1. For PD, the seed nodes TFIIH p34 subunit and TFB5 show higher and lower expression respectively.
GO:0009452: 7-methylguanosine RNA capping	Only two gene products in common: POLR2B and GATA-1. For PD, the seed nodes TFIIH p34 subunit and TFB5 show higher and lower expression respectively.
GO:0014054: positive regulation of gamma-aminobutyric acid secretion	The overlap of this GO term with the subnetworks for PD and HGPS only contains nodes from the seed node neighborhood, and the overlaps have only one gene product in common (Serotonin receptor).
GO:0007166: cell surface receptor signaling pathway	The cell surface receptors partially differ between the subnetworks for PD and HGPS (PD: JAK-STAT receptor, TCF receptors; HGPS: NOTCH1receptor, TGF-beta receptors, JAK-STAT receptor, TCF receptors).

References

[1] Dijkstra, A.A., Ingrassia, A., de Menezes, R.X., van Kesteren, R.E., Rozemuller, A.J., Heutink, P., van de Berg, W.D.: Evidence for immune response, axonal dysfunction and reduced endocytosis in the substantia nigra in early stage parkinson's disease. PLoS One **10**(6), 0128651 (2015)

[2] Riley, B.E., Gardai, S.J., Emig-Agius, D., Bessarabova, M., Ivliev, A.E., Schüle, B., Alexander, J., Wallace, W., Halliday, G.M., Langston, J.W., et al.: Systems-based analyses of brain regions functionally impacted in parkinson's disease reveals underlying causal mechanisms. PLoS One **9**(8) (2014)

[3] Zheng, B., Liao, Z., Locascio, J.J., Lesniak, K.A., Roderick, S.S., Watt, M.L., Eklund, A.C., Zhang-James, Y., Kim, P.D., Hauser, M.A., *et al.*: Pgc-1α, a potential therapeutic target for early intervention in parkinson's disease. Science translational medicine **2**(52), 52–73 (2010)

[4] Zhang, Y., James, M., Middleton, F.A., Davis, R.L.: Transcriptional analysis of multiple brain regions in parkinson's disease supports the involvement of specific protein processing, energy metabolism, and signaling pathways, and suggests novel disease mechanisms. American Journal of Medical Genetics Part B: Neuropsychiatric Genetics **137**(1), 5–16 (2005)

[5] Moran, L.B., Duke, D., Deprez, M., Dexter, D., Pearce, R., Graeber, M.: Whole genome expression profiling of the medial and lateral substantia nigra in parkinson's disease. Neurogenetics **7**(1), 1–11 (2006)

[6] Lesnick, T.G., Papapetropoulos, S., Mash, D.C., Ffrench-Mullen, J., Shehadeh, L., de Andrade, M., Henley, J.R., Rocca, W.A., Ahlskog, J.E., Maraganore, D.M.: A genomic pathway approach to a complex disease: axon guidance and parkinson disease. PLoS genetics **3**(6), 98 (2007)

[7] Simunovic, F., Yi, M., Wang, Y., Macey, L., Brown, L.T., Krichevsky, A.M., Andersen, S.L., Stephens, R.M., Benes, F.M., Sonntag, K.C.: Gene expression profiling of substantia nigra dopamine neurons: further insights into parkinson's disease pathology. Brain **132**(7), 1795–1809 (2008)

[8] Csoka, A.B., English, S.B., Simkevich, C.P., Ginzinger, D.G., Butte, A.J., Schatten, G.P., Rothman, F.G., Sedivy, J.M.: Genome-scale expression profiling of hutchinson–gilford progeria syndrome reveals widespread transcriptional misregulation leading to mesodermal/mesenchymal defects and accelerated atherosclerosis. Aging cell **3**(4), 235–243 (2004)

[9] Marji, J., O'Donoghue, S.I., McClintock, D., Satagopam, V.P., Schneider, R., Ratner, D., Worman, H.J., Gordon, L.B., Djabali, K.: Defective lamin a-rb signaling in hutchinson-gilford progeria syndrome and reversal by farnesyltransferase inhibition. PLoS One **5**(6), 11132 (2010)

[10] Plasilova, M., Chattopadhyay, C., Ghosh, A., Wenzel, F., Demougin, P., Noppen, C., Schaub, N., Szinnai, G., Terracciano, L., Heinimann, K.: Discordant gene expression signatures and related phenotypic differences in lamin a-and a/c-related hutchinson-gilford progeria syndrome (hgps). PloS one **6**(6), 21433 (2011)

[11] Berchtold, N.C., Cribbs, D.H., Coleman, P.D., Rogers, J., Head, E., Kim, R., Beach, T., Miller, C., Troncoso, J., Trojanowski, J.Q., *et al.*: Gene expression changes in the course

of normal brain aging are sexually dimorphic. Proceedings of the National Academy of Sciences **105**(40), 15605–15610 (2008)

[12] Hokama, M., Oka, S., Leon, J., Ninomiya, T., Honda, H., Sasaki, K., Iwaki, T., Ohara, T., Sasaki, T., LaFerla, F.M., *et al.*: Altered expression of diabetes-related genes in alzheimer's disease brains: the hisayama study. Cerebral cortex **24**(9), 2476–2488 (2014)

[13] Liang, W.S., Dunckley, T., Beach, T.G., Grover, A., Mastroeni, D., Walker, D.G., Caselli, R.J., Kukull, W.A., McKeel, D., Morris, J.C., *et al.*: Gene expression profiles in anatomically and functionally distinct regions of the normal aged human brain. Physiological genomics **28**(3), 311–322 (2007)

[14] Raskin, L., Fullen, D.R., Giordano, T.J., Thomas, D.G., Frohm, M.L., Cha, K.B., Ahn, J., Mukherjee, B., Johnson, T.M., Gruber, S.B.: Transcriptome profiling identifies hmga2 as a biomarker of melanoma progression and prognosis. Journal of Investigative Dermatology **133**(11), 2585–2592 (2013)

[15] Riker, A.I., Enkemann, S.A., Fodstad, O., Liu, S., Ren, S., Morris, C., Xi, Y., Howell, P., Metge, B., Samant, R.S., *et al.*: The gene expression profiles of primary and metastatic melanoma yields a transition point of tumor progression and metastasis. BMC medical genomics **1**(1), 13 (2008)

[16] Straniero, L., Guella, I., Cilia, R., Parkkinen, L., Rimoldi, V., Young, A., Asselta, R., Soldà, G., Sossi, V., Stoessl, A.J., *et al.*: Dnajc12 and dopa-responsive nonprogressive parkinsonism. Annals of neurology **82**(4), 640–646 (2017)

[17] Fan, Y., Yang, Z.-h., Li, F., Hu, X.-c., Yue, Y.-w., Yang, J., Liu, Y.-t., Liu, H., Wang, Y.-l., Shi, C.-h., *et al.*: Dnajc12 mutation is rare in chinese han population with parkinson's disease. Neurobiology of aging **68**, 159–1 (2018)

[18] Dil Kuazi, A., Kito, K., Abe, Y., Shin, R.-W., Kamitani, T., Ueda, N.: Nedd8 protein is involved in ubiquitinated inclusion bodies. The Journal of Pathology: A Journal of the Pathological Society of Great Britain and Ireland **199**(2), 259–266 (2003)

[19] Choo, Y.S., Vogler, G., Wang, D., Kalvakuri, S., Iliuk, A., Tao, W.A., Bodmer, R., Zhang, Z.: Regulation of parkin and pink1 by neddylation. Human molecular genetics **21**(11), 2514–2523 (2012)

[20] Nalls, M.A., Blauwendraat, C., Vallerga, C.L., Heilbron, K., Bandres-Ciga, S., Chang, D., Tan, M., Kia, D.A., Noyce, A.J., Xue, A., *et al.*: Identification of novel risk loci, causal insights, and heritable risk for parkinson's disease: a meta-analysis of genome-wide association studies. The Lancet Neurology **18**(12), 1091–1102 (2019)

[21] Chen, Y.P., Song, W., Huang, R., Chen, K., Zhao, B., Li, J., Yang, Y., Shang, H.-F.: Gak rs1564282 and dgkq rs11248060 increase the risk for parkinson's disease in a chinese population. Journal of Clinical Neuroscience **20**(6), 880–883 (2013)

[22] Pankratz, N., Wilk, J.B., Latourelle, J.C., DeStefano, A.L., Halter, C., Pugh, E.W., Doheny, K.F., Gusella, J.F., Nichols, W.C., Foroud, T., *et al.*: Genomewide association study for susceptibility genes contributing to familial parkinson disease. Human genetics **124**(6), 593–605 (2009)

[23] Nagle, M.W., Latourelle, J.C., Labadorf, A., Dumitriu, A., Hadzi, T.C., Beach, T.G., Myers, R.H.: The 4p16. 3 parkinson disease risk locus is associated with gak expression and genes involved with the synaptic vesicle membrane. PloS one **11**(8) (2016)

[24] Supek, F., Bošnjak, M., Škunca, N., Šmuc, T.: Revigo summarizes and visualizes long lists of gene ontology terms. PloS one **6**(7) (2011)
